# Mitochondria Deregulations in Cancer Offer Several Potential Targets of Therapeutic Interventions

**DOI:** 10.3390/ijms241310420

**Published:** 2023-06-21

**Authors:** Clara Musicco, Anna Signorile, Vito Pesce, Paola Loguercio Polosa, Antonella Cormio

**Affiliations:** 1 Institute of Biomembranes, Bioenergetics and Molecular Biotechnologies (IBIOM), CNR, 70126 Bari, Italy; 2 Department of Translational Biomedicine and Neuroscience, University of Bari “Aldo Moro”, 70124 Bari, Italy; 3 Department of Biosciences, Biotechnologies and Environment, University of Bari “Aldo Moro”, 70125 Bari, Italy; 4 Department of Precision and Regenerative Medicine and Ionian Area, University of Bari “Aldo Moro”, 70124 Bari, Italy

**Keywords:** cancer therapy, targeting mitochondria, mitochondrial inhibitors, ROS, mitochondrial drug delivery

## Abstract

Mitochondria play a key role in cancer and their involvement is not limited to the production of ATP only. Mitochondria also produce reactive oxygen species and building blocks to sustain rapid cell proliferation; thus, the deregulation of mitochondrial function is associated with cancer disease development and progression. In cancer cells, a metabolic reprogramming takes place through a different modulation of the mitochondrial metabolic pathways, including oxidative phosphorylation, fatty acid oxidation, the Krebs cycle, glutamine and heme metabolism. Alterations of mitochondrial homeostasis, in particular, of mitochondrial biogenesis, mitophagy, dynamics, redox balance, and protein homeostasis, were also observed in cancer cells. The use of drugs acting on mitochondrial destabilization may represent a promising therapeutic approach in tumors in which mitochondrial respiration is the predominant energy source. In this review, we summarize the main mitochondrial features and metabolic pathways altered in cancer cells, moreover, we present the best known drugs that, by acting on mitochondrial homeostasis and metabolic pathways, may induce mitochondrial alterations and cancer cell death. In addition, new strategies that induce mitochondrial damage, such as photodynamic, photothermal and chemodynamic therapies, and the development of nanoformulations that specifically target drugs in mitochondria are also described. Thus, mitochondria-targeted drugs may open new frontiers to a tailored and personalized cancer therapy.

## 1. Introduction

Rapid proliferation is a characteristic of tumor cells, and metabolic reprogramming is a hallmark of many cancer cells in support of large-scale biosynthetic programs [1]. For this purpose, cancer cells actively metabolize glucose, increasing the glycolytic rate, and produce an excess of lactate, even in the presence of oxygen (Warburg effect) [2], in order to increase the NAD^+^/NADH cell ratio, which is essential to promote glycolysis. For many years, it was the opinion that the Warburg effect could be the consequence of mitochondrial dysfunction. Emerging evidence indicates that tumor metabolic reprogramming is much more complex than that theorized by Warburg and that cancer cells are able to change their metabolism in a hybrid metabolic phenotype, equilibrating glycolysis and the mitochondrial oxidative phosphorylation (OXPHOS) system. Mitochondrial metabolism is essential for many cancer cells not only for the great production of ATP, but also because it provides the building blocks for biosynthetic programs [3].

Tumor cells not only consume a large amount of glucose but also metabolize fatty acids and amino acids, in particular glutamine, since they represent the main source of carbon for ATP production and biosynthesis [4]. Glutamine generates glutamate, and its conversion into alpha ketoglutarate (α-KG) is very important for supplying the tricarboxylic acid (TCA) cycle with the anabolic intermediates needed for fatty acid, amino acid, or porphyrin biosynthesis. These metabolic pathways involve mitochondria.

Mitochondria are cellular organelles that have their own genome (mtDNA) and are surrounded by two membranes. The outer and inner membranes limit the mitochondrial intermembrane and matrix space, respectively. The outer membrane is permeable, allowing the diffusion of molecules via the porin voltage dependent anion channel; the inner membrane is less permeable and contains the respiratory chain complexes.

Mitochondria are crucial in eukaryotic cells as they produce most of the ATP and reactive oxygen species (ROS), buffer intracellular calcium, and regulate apoptosis as well as signal transduction [5,6].

Many cellular aspects involving mitochondria have been found to be altered in cancer cells, such as mitochondrial metabolic pathways, mtDNA mutations, respiratory chain complex activities, redox balance, cell apoptosis, mitophagy, dynamics, and the signal transduction pathway [7,8,9,10].

Therefore, drugs causing mitochondrial destabilization are considered a promising therapeutic approach for cancer treatment, particularly in tumors in which mitochondrial respiration is the predominant energy source. These anticancer agents are also called mitocans. Mitocans are molecules that may induce mitochondrial dysfunction leading to cancer cell death.

Mitocans can be divided into three main groups: (1) molecules targeting mitochondrial metabolic pathways, such as the Krebs cycle, glutamine metabolism, fatty acid oxidation, oxidative phosphorylation (OXPHOS), and heme biosynthesis; (2) molecules targeting mitochondrial homeostasis, such as mitochondrial biogenesis, apoptosis, dynamics, mitophagy, proteostasis, and redox balance; (3) molecules that, through photodynamic, photothermal and chemodynamic processes, can produce mtROS and heat to induce mitochondrial damage. In this review, we provide an overview of the most known mitochondria-targeting agents possibly useful in cancer therapy, as well as an update of novel strategies to specifically deliver drugs to mitochondria.

## 2. Molecules Targeting Mitochondrial Metabolic Pathways

### 2.1. Drugs Targeting the Krebs Cycle

The tricarboxylic acid (TCA) cycle, or Krebs cycle, represents an important part of aerobic respiration. The Krebs cycle takes place in the mitochondrial matrix where acetyl-CoA is oxidized into two 2 CO_2_, with 3 NADH, 2 FADH, and 1 GTP also produced. Then, NADH and FADH2 donate electrons to the respiratory chain complexes to produce ATP. Many of the intermediates upon which the cycle depends also give rise to pathways leading to important compounds, such as fatty acids, amino acids, and porphyrins.

Mutations in the TCA enzymes, such as succinate dehydrogenase (SDH), fumarate hydratase (FH), and isocitrate dehydrogenase (IDH), have been reported in different cancers and are also responsible for the induction of aerobic glycolysis. SDH and FH mutations are loss-of-function mutations, causing the accumulation of succinate and fumarate, respectively, leading to a range of subsequent intracellular metabolic changes that induces a glycolytic shift [11,12]. On the contrary, IDH mutations result in a gain of function, converting α-KG into the oncometabolite 2-hydroxyglutarate (2-HG) that inhibits α-ketoglutarate–dependent enzymes, resulting in tumorigenesis. Clinical studies demonstrated that inhibitors of IDH, namely Ivosidenib and Vorasidenib, reducing the production of the 2-HG, were also able to induce remissions in patients with acute myeloid leukemia [13] and to increase survival in patients with glioma, respectively [14]. 

### 2.2. Drugs Targeting Fatty Acid Oxidation

In some cancers, elevated fatty acid oxidation (FAO) also provides high ATP levels to sustain proliferation. In these cells, a simultaneous increase in fatty acid synthesis and FAO occurs through changes in mitochondrial and histone acetylation [15]. FAO takes place in the mitochondria matrix; therefore, for a fatty acid to enter the mitochondria, the activity of carnitine palmitoyltransferase 1 enzyme (CPT-1) is required [16]. CPT-1 is upregulated in different cancers, and the suppression of CPT-1 can lead to cell death and reduces cancer progression [17,18].

A CPT-1 inhibitor, Etomoxir, has been shown to be effective in preventing tumor progression in glioblastoma and glioma [19,20]. However, Etomoxir at high doses induces toxicity, therefore, its clinical use has ceased. ST1326, a reversible inhibitor of CPT-1, has been shown to inhibit FAO in leukemia cells with associated growth arrest, mitochondrial damage and apoptosis [21]. A more specific inhibitor of CPT-1, Perhexiline [22], a drug used to treat some cardiovascular diseases, kills colorectal cancer cells as either monolayers or spheroids, and patient-derived organoids from primary and metastatic sites [23].

### 2.3. Drugs Targeting Glutamine Metabolism

Glutamine is the most prevalent amino acid in blood and is involved in the synthesis of nucleic acids, amino acids and glutathione. Glutamine is converted into glutamate by glutaminase (GLS), which is in turn converted into α-KG by glutamate dehydrogenase (GDH); in this way, it can enter the Krebs cycle as a source of TCA cycle intermediates.

Moreover, α-KG is also produced from glutamate by mitochondrial Glutamate-Oxaloacetate transaminase (GOT1) and Glutamate-Pyruvate transaminase (GPT2). Increased glutamine metabolism is a hallmark of many cancer types [24]. Therefore, the inhibition of glutamine uptake by the glutamine transporter (ASCT2), as well as the inhibition of glutaminase, GDH, GOT1 and GPT2, seem to all be good strategies to decrease the replenishment of intermediates in the TCA cycle and, consequently, cancer cell proliferation.

Pharmacological inhibition of ASCT2-dependent glutamine transport leads to an antitumor effect in lung, breast, and colorectal cancer cell lines [25]. Moreover, several small-molecules, such as BPTES, UPGL00004, and CB-839, have been reported as inhibitors of GLS, and they exhibit remarkable antiproliferation activity [26,27]. GDH is upregulated in human lung and breast cancers and the inhibition of this enzyme with Propylselen reduces cancer cell growth in liver and lung carcinomas [28]. Moreover, several inhibitors of GOT1 and GPT2, likely Hydralazine hydrochloride, Aspulvinone O, Oxamate and Aminooxyacetate, were reported to reduce growth in different cancer lines [29,30,31,32].

### 2.4. Drugs Targeting the Oxidative Phosphorylation (OXPHOS) System

The OXPHOS is located in the inner mitochondrial membrane cristae and it is composed of large protein complexes called NADH Ubiquinone-oxidoreductase (Complex I), succinate dehydrogenase (Complex II), cytochrome c reductase (Complex III), cytochrome c oxidase (Complex IV), ATP synthase (Complex V), and two freely mobile electron transfer carriers, ubiquinone and cytochrome c. Three of these complexes (Complexes I, CIII, CIV) act as proton pumps and contribute to the formation of an electrochemical proton gradient across the inner mitochondrial membrane. This gradient drives ATP synthesis by complex V (ATP synthase) via an oxidative phosphorylation process [33]. Electrons donated by NADH and FADH2 to the respiratory chain complexes flow to O_2_, which is the last electron acceptor. Some of the electrons are leaked and generate ROS.

**Figure 1:**
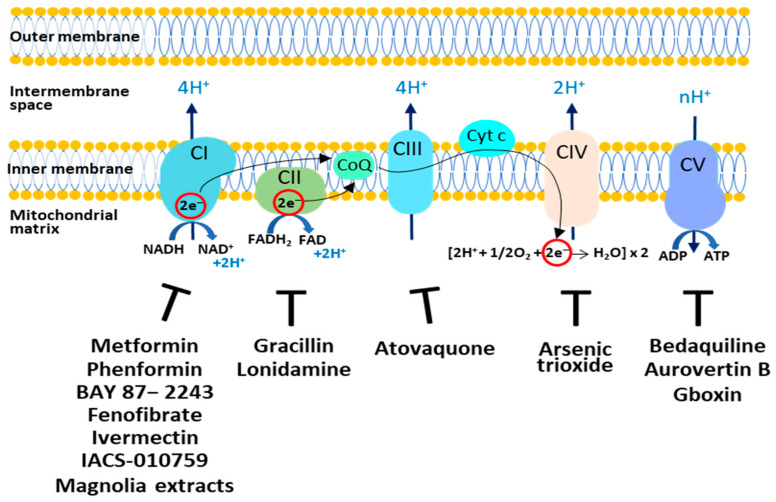
Targeting the OXPHOS system in cancer cells. The respiratory complexes and their respective inhibitors discussed in this review are shown. CI: Complex I; CII: Complex II; CIII: Complex III; CIV: Complex IV; CV: Complex V; CoQ: Coenzyme Q; Cyt c: Cytochrome c.

The OXPHOS plays a controversial role in tumor growth and survival. In fact, it is reported that in cancer cells OXPHOS can be downregulated or upregulated, controlling tumor progression, aggressiveness, and resistance to chemotherapy [34]; thus, targeting OXPHOS represents an interesting therapeutic opportunity. Synthetic and natural compounds are able to inhibit oxidative phosphorylation acting on the five different complexes (Figure 1). Some of these drugs are approved by the FDA and are currently used in nontumor therapies.

Complex I represents a very interesting target since it is overexpressed in various cancer types and its overexpression is also related to tumorigenesis and patient prognosis [35]. Among the various drugs that affect Complex I, the most studied is Metformin [36], the widely prescribed drug used for type 2 diabetes. Recent studies indicate that Metformin acts as a cancer preventive agent by reducing the relative cancer risk [37], and as an inhibitor of cancer cell proliferation [38]. The mechanisms that may explain its anticancer properties are still not fully understood. First, Metformin at high doses inhibits CI, causing bioenergetic stress in cancer cells and rendering them dependent on glycolysis for ATP production [39,40]. Metformin, via the inhibition of Complex I and a decrease in ATP synthesis, increases the AMP:ATP ratio and activates Adenosine Monophosphate–Activated Protein Kinase (AMPK) via phosphorylation. Activated AMPK promotes a phosphorylation cascade and induces the inhibition of mTOR, a serine threonine kinase that is the catalytic subunit of mTOR complex 1 (mTORC1) and complex 2 (mTORC2), which are downstream components of the cell cycle regulating pathway PI3K/AKT. Inhibition of mTOR promotes catabolism and cell cycle arrest in different cancer cells [40]. Moreover, Metformin may act indirectly by reducing blood insulin and IGF1 levels. The PI3K/AKT-mTOR pathway is directly downstream of insulin and IGF1 receptor signaling [41]. Interestingly, clinical trial data show that Black cancer patients respond better to Metformin than white cancer patients [42]. This behavior can be explained by considering that cancer cells from Black patients show more mitochondria and upregulation of genes associated with OXPHOS [43].

Phenformin, a precursor of Metformin, has shown great anticancer activity in neuroblastoma and pancreatic cancer cells [44,45]. BAY 87−2243 depolarizes the mitochondrial membrane potential (Δψ), increases cellular ROS levels by blocking mitochondrial complex I [46] and induces melanoma cell death. Fenofibrate, a drug used to treat lipid metabolism, displays anti-tumour activity in glioblastoma and gastric cancer [47,48]. Ivermectin, an antiparasitic drug, reduces proliferation in myeloid leukemia and renal cancer by inhibiting mitochondrial Complex I activity [49,50]. ACS-010,759 is a potent oral selective inhibitor of mitochondrial Complex I. Treatment with IACS-010759 inhibits proliferation and induces apoptosis in models of brain cancer and acute myeloid leukemia (AML) where mitochondrial respiration is the predominant energy source [51]. Honokiol and Magnolol (magnolia extracts) inhibit mitochondrial Complex I, inducing ROS increase, AMPK activation and, finally, a decrease in tumor proliferation [52].

To limit the ability of cancer cells to generate ATP via OXPHOS, CΙI activity can also be inhibited. The natural compound Gracillin has been reported to disrupt respiratory CII activity; in breast cancer cell lines, it inhibits tumor growth by inducing apoptosis [53]. In addition, Lonidamine was found to have antitumor activity in several cancer cells by inhibiting CII and hexokinase activity; thus, leading to decreased energy production and apoptosis [54,55].

For the inhibition of complex III, the analogue of ubiquinone, Atovaquone, has raised much interest. This drug, approved by the FDA as an antimalaria drug, is well-tolerated and represents a competitive inhibitor of mitochondrial Complex III by displacing ubiquinol at the active site of the cytochrome bc1 complex. Atovaquone inhibits mitochondrial respiration and reduces tumor proliferation in vitro and in vivo in hypopharyngeal, colorectal and lung carcinoma cells [56].

An inhibitor of Complex IV, named Arsenic trioxide (ATO), has been reported to increase the ROS level, which caused the death of cancer cells [57,58]. However, this drug shows toxicity and several side effects.

Different molecules, by targeting on Complex V, have shown anticancer activity. Bedaquiline, an FDA-approved antibiotic used for the treatment of multidrug-resistant pulmonary tuberculosis, reduces OXPHOS activity in tumor cells by inhibiting Complex V. Moreover, it has been shown that Bedaquiline downregulates the expression of the Complex V subunit ATP5F1C in vitro and prevents in vivo spontaneous metastasis [59].

The natural compound Aurovertin B is an inhibitor of Complex V and reduces cancer cell proliferation [60]. Benzimidazolinium-gboxin (Gboxin) is a small positive charge molecule that inhibits Complex V and reduces the growth of human glioblastoma cells [61].

### 2.5. Drugs Targeting Heme Biosynthesis

The heme group is a molecule that binds oxygen; it is present in several proteins, including those of Complex II, III and IV of the mitochondrial respiratory chain. Increased heme biosynthesis and uptake have been found in several tumors, suggesting that heme uptake and incorporation into oxygen-utilizing heme proteins is necessary for cancer cell growth [62,63,64].

Succinylacetone, a potent inhibitor of 5-aminolevulinate dehydratase (ALA-D), which is the second enzyme in the heme biosynthetic pathway, shows potential anticancer activity, inducing heme depletion and mitochondrial dysfunction [65,66,67].

Cyclopamine tartrate (CycT) induces cell death in lung tumor cells and tumor xenograft mouse models by reducing the activity of aminolevulinate synthase 1 (ALAS1), the first rate-limiting enzyme in heme biosynthesis [68]. Therefore, heme-targeting drugs inhibit tumor growth by decreasing oxygen consumption and ATP generation. 

**Figure 2:**
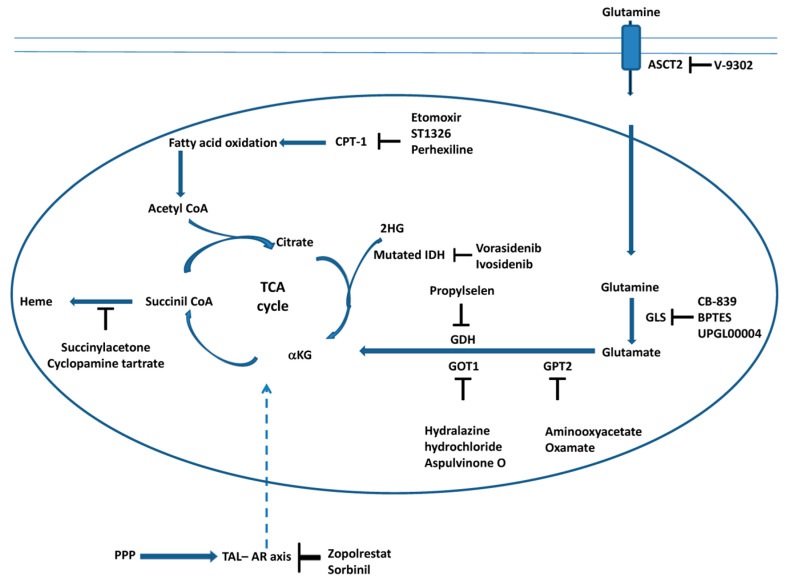
Targeting the TCA cycle, glutaminolysis, fatty acid oxidation and heme biosynthesis in cancer cells. The scheme illustrates the main steps of the TCA, glutaminolysis, fatty acid oxidation and heme biosynthesis inhibited by the molecules discussed in this review. Moreover, the interaction between the cytosolic pentose phosphate pathway (PPP) and the TCA cycle during carcinogenesis is also reported (see Section 3.6). The transaldolase (TAL)–aldose reductase (AR) axis connects the PPP and TCA cycle (dashed arrow). Inhibitors of AR, which affect mitochondrial metabolism, are also reported (see Section 3.6).

In conclusion, the connection between metabolic pathways, such as the TCA cycle, glutaminolysis, fatty acid oxidation and heme biosynthesis (Figure 2), is fundamental to the survival and proliferation of cancer cells. Glutaminolysis and fatty acid oxidation supplies the TCA cycle with anabolic intermediates; one of these intermediates, succinyl CoA, is a substrate for heme biosynthesis. Therefore, the use of specific inhibitors of these metabolic pathways, as reported in Figure 2, represents a good strategy for killing cancer cells.

The drugs targeting mitochondrial metabolic pathways, discussed in previous paragraphs, are presented in Table 1. The molecular targets and the anti-tumor effects of these molecules are also reported.

## 3. Molecules Targeting Mitochondrial Homeostasis

### 3.1. Drugs Targeting Mitochondrial Biogenesis

Replication and maintenance of the mtDNA depends on the DNA polymerase γ that is responsible for mtDNA replication and repair. Therefore, DNA polymerase γ could represent a target for anticancer drug development. Vitamin K3 or Menadione targets mtDNA by inhibiting the activity of mtDNA polymerase γ and inducing the apoptosis of cancer cells [69]. Additionally, inhibitors of the mitochondrial DNA-dependent RNA polymerase (POLRMT) were recently identified as new anticancer compounds. These molecules, named IMT1 and IMT1B, cause selective cancer cell apoptosis by affecting the transcription of mitochondrially-encoded OXPHOS subunits [70].

The master regulator of mitochondrial biogenesis is the nuclear transcriptional PPAR γ co-activator 1α (PGC-1α). It targets many nuclear receptors and transcription factors, in particular, peroxisome proliferator activated receptors (PPARs), the estrogen related receptor (ERRα), and nuclear respiratory factor 1 and 2 (NRF1/NRF2) [71]. These factors stimulate the expression of a large number of nuclear genes involved in mitochondrial respiration, FAO and mtDNA replication and transcription. PGC-1α upregulation has been reported in different cancers associated with increased OXPHOS [10,72,73]. However, PGC-1α in breast cancer and osteosarcoma acts as a tumor suppressor [74,75]. Specific inhibitors of PGC-1α with an anticancer effect have not been developed, yet. Nevertheless, inhibitors targeting PGC-1α-related factors, such as ERRα, show good anticancer activity [76]. Inhibitors of ERRα, such as XCT790, Gemcitabine, and SLU-PP-1072, decrease mitochondrial biogenesis in cancer cells, reducing their proliferation and increasing their sensitivity to chemotherapeutic agents both in vitro and in vivo [77,78,79].

An important target of mitochondrial biogenesis regulation is mTOR, which regulates cell proliferation, energy homeostasis, the mitochondrial transcription program, and biogenesis, through the YY1–PGC-1α axis [80]. Rapamycin, an FDA-approved drug for the prophylaxis of organ rejection in renal transplantation, was the first mTOR inhibitor with anticancer potential [81]. Unfortunately, its low solubility and consequent pharmacokinetic problems have limited its clinical use. Analogues of Rapamycin, named Rapalogs, such as Temsirolimus, Sirolimus, and Everolimus, are selective inhibitors of mTOR catalytic activity [For review, 81]. Clinical studies show that Temsirolimus is active in different cancers [82,83]. Temsirolimus has already been approved by the FDA for treating advanced renal cell cancer [82]. Moreover, XL388, Torin 1 and 2, a new class of mTOR inhibitors, suppress tumor cell proliferation and migration in glioblastoma [84].

### 3.2. Drugs Targeting Apoptosis by Bcl-2 Family Proteins

Mitochondria are the main pro-apoptotic targets of ROS, which can induce the opening of the permeability transition pore (PTP) complex located in the mitochondrial outer and inner membranes. PTP opening induces the release of calcium ions, cytochrome c, apoptosis-inducing factor AIF and other factors, causing caspase activation and, finally, apoptosis [85]. Proteins of the Bcl-2 family are regulators of apoptosis and are both pro- and antiapoptotic members. The antiapoptotic proteins, B-cell lymphoma-2 (Bcl-2) and B-cell lymphoma-extra Large (Bcl-xL), are located in the outer mitochondrial membrane and inhibit PTP opening and the release of cytochrome c. The cytoplasmic pro-apoptotic proteins Bax, Bad, Bid and Bim translocate to the mitochondria upon receiving a death signal and promote PTP opening [85]. The antiapoptotic Bcl-2 family proteins are found to be overexpressed in cancer cells [86]. Bcl-2 consists of four conserved domains (BH4, BH3, BH1, and BH2), which differentiate it from other Bcl-2 family members. Most Bcl-2 inhibitors, Venetoclax, ABT-737, Navitoclax, ABT-263, Obatoclax, AT-101 and Oblimersen, are BH3-mimetics, which means that they can bind to the BH3 domain of Bcl-2, impeding the binding of Bim to Bcl-2. As a consequence, free Bim can activate Bak/Bax on the surface of mitochondria, inducing PTP opening and the death of cancer cells [87,88,89]. Preclinical studies demonstrated that these inhibitors were able to inhibit tumor growth in vivo and in vitro; however, clinical studies are needed to prove their real efficacy [90].

### 3.3. Drugs Targeting Mitochondrial Dynamics

Mitochondria are highly dynamic organelles; they continuously fuse via a fusion process and divide via a fission process. Fusion is regulated by mitofusin (Mfn1 and Mfn2) proteins in the outer mitochondrial membrane and by the optic atrophy 1 (Opa1) protein in the inner mitochondrial membrane. Fission is mediated by the dynamin-related protein 1 (Drp1) and the mitochondrial fission1 protein (Fis1) [91]. Alterations in mitochondrial dynamics have been implicated in an increasing number of diseases including cancer [10,92]. An increased expression of Drp1 and a downregulation of Mfn-2 have been observed in endometrial cancer, resulting in an imbalance of mitochondrial dynamics towards fission [93]. The selective inhibitor of Drp1, Mdivi-1, decreased fission, oxidative metabolism, and impaired cell proliferation in different cancer cell lines [94,95]. Moreover, two Ellipticine analogues, namely Drpitor1 and Drpitor1a, displayed a selective inhibitory activity against Drp1, which impaired mitochondrial fission; this resulted in the inhibition of cancer cell proliferation and apoptosis in both cancer cell cultures and xenograft mouse models [96]. In breast cancer stem cells, an increased expression of Mitofusin1 was found and the administration of AZD5363 suppressed Mitofusin1 expression and induced apoptosis [97].

### 3.4. Drugs Targeting Mitophagy

The machinery regulating mitochondrial dynamics is highly integrated with mitophagy as part of the mitochondrial quality control process. After fission, functional daughter mitochondria can undergo fusion, whereas non-functional mitochondria are targeted by mitophagic proteins to degradation [98]. Mitophagy plays a double-edged role in cancer: a normal level of mitophagy is a defensive mechanism that eliminates damaged mitochondria and promotes cell survival, whereas excessive mitophagy may impair mitochondrial function and induce apotosis. Therefore, the induction or inhibition of mitophagy may represent a good strategy in cancer treatment [99]. In malignant gliomas, ceramide can increase the expression of the mitophagic protein BNIP3, inducing mitophagy and, finally, cancer cell death [100]. Dihydroergotamide tartrate activates the PINK1/Parkin-mediated mitophagy pathway promoting cell death in lung cancer cells [101]. Moreover, inhibition of mitophagy can be also an effective strategy for treating cancer. For example, melatonin can inhibit mitophagy by downregulating Parkin, thus leading to apoptosis of cervical cancer cells [102].

### 3.5. Drugs Targeting Mitochondrial Proteostasis

#### 3.5.1. Drugs Targeting Mitochondrial Protein Synthesis

Mitochondrial protein synthesis represents a promising target in cancer treatment. Tigecycline, a Tetracycline derivative used as an antibiotic for the prevention of malaria, targets the small subunit 28S and, as a consequence, inhibits mitochondrial protein translation. The impairment of mitochondrial protein synthesis leads to decreased mitochondrial ATP production, slowing cell proliferation in several cancer cell lines [103]. However, the use of antibiotics may lead to the development of antibiotic resistance; furthermore, this could have a pro-cancer effect by disrupting intestinal microbiota, which are protective against neoplastic transformation [104]. Therefore, to reduce the side effect of antibiotics in cancer treatments, it is preferable to associate these with probiotics and prebiotics.

#### 3.5.2. Drugs Targeting Mitochondrial Proteases

Cancer cell growth is associated with stress conditions that may cause protein damage. Mitochondrial proteases, namely Caseinolytic protease P (ClpP), Lon protease, HtrA2 and OMA, are important for protein maintenance since they degrade misfolded, oxidized and aged proteins. The first two proteases are present in the mitochondrial matrix, and the latter in the inner mitochondrial membrane [105]. These proteases degrade proteins located in all mitochondrial compartments including the subunits of the respiratory complexes and translocases.

ClpP is a mitochondrial serine protease, which binds the chaperone protein AAA+ ClpX resulting in a complex called ClpXP. The proteolysis occurs in two steps: ClpX binds and partially unfolds the target proteins in an ATP-dependent process, then delivers the proteins to the ClpP proteolytic chamber for degradation [106,107]. ClpP is upregulated in primary and metastatic human tumors, supports tumor cell proliferation, and its overexpression desensitizes cells to cisplatin [108]. Interestingly, small modulators of ClpP activity, both activators and inhibitors, are able to impair oxidative phosphorylation in cancer cells and to induce apoptosis. Trans-ß-lactones and Phenyl ester compounds (AV167, TG42 and TG43) inhibit human ClpP proteolytic activity and induce apoptosis, in AML cell lines and hepatocyte-derived carcinoma cells, respectively [109,110].

Wong et al. [111] demonstrated that Acyldepsipeptides (ADEP-41), a ClpP protease activator, destroys OXPHOS function and induces apoptosis in kidney, cervical, osteosarcoma and neuroblastoma cells.

The imipridone molecule ONC201 is an activator of ClpP with antiproliferative and pro-apoptotic effects in numerous solid tumors and hematological malignancies. ClpP activation causes the degradation of mitochondrial proteins, including the subunits of the respiratory complexes, leading to the decreased activity of Complexes I, II and IV, and increased ROS production [112]. ONC201, and the more potent analogue ONC212, induce lethality in leukemia and lymphoma cell lines, as well as in primary AML cells, without affecting non-tumor cells [113].

LonP is a homohexamer that shows three different activities (proteolysis, chaperone activity, binding of mtDNA) [114]. Moreover, it regulates the Krebs cycle, oxidative phosphorylation, steroid and heme biosynthesis, and glutamine production. LonP1 is upregulated in many cancers, such as lung, breast, colon, prostate, stomach, glioma, pancreatic, endometrial, melanoma cancer and leukemia [115,116]. Gibellini et al. demonstrated that LonP1 inhibition increases mitochondrial ROS accumulation, and triggers oxidative stress and apoptosis in cancer cells, while LonP1 overexpression reduces cell death [117]. Cyano-3,12-dioxooleana-1,9-dien-28-oic acid (CDDO) and a derivative of CDDO (CDDO-ME) block the LonP1 protease activity in vitro and show efficient antitumor activity at the cellular level, inducing cell death in hepatocarcinoma, colon, and breast cancer cells without toxicity to normal cells [117]. Obtusilactone A and Sesamin, which inhibit LonP1 protease activity, were shown to reduce cancer cell proliferation and migration in lung cancer cells (A549 and H1299), with low cytotoxicity in healthy cells [118].

High-temperature requirement protease A2 (HtrA2) (also called OMI) is a serine peptidase located in the inner mitochondrial membrane; it possesses proteolytic activity in the trimeric form and does not require the presence of ATP [119]. HtrA2 seems to be a pro-apoptotic protein as its over-expression triggers cell death. HtrA2 is activated when it binds the apoptosis inhibitor IAP; it then cleaves IAP and decreases caspase inhibition. HtrA2 also cleaves antiapoptotic proteins, such as Pea-15, DUSP9, GRIM 19, and Hax-1, which are involved in caspase-independent apoptosis [120]. However, the role of HtrA2 is not fully understood, since an increased or decreased expression of HtrA2 was reported in different cancers [120].

OMA1 is a conserved metalloendopeptidase that exerts its physiological effects in an ATP-independent manner [121]. OMA1 induces OPA1 hydrolysis leading to cytochrome c release followed by mitochondrial swelling and rupture and, finally, apoptosis [122]. Lower expression of OMA1 correlates with poor overall survival in breast cancer patients. Silencing OMA1 in vitro in metastatic breast cancer cells increases cell proliferation and induces an epithelial–mesenchymal transition (EMT) [123].

The proteases HtrA2 and OMA1 might be new targets in cancer therapy; however, at the moment, no studies with specific modulators of these enzymes are available.

### 3.6. Drugs Targeting the Cellular Redox Balance

Mitochondria are one of the major sources of ROS, which have an important role in several cellular events, such as cellular proliferation, differentiation and migration. Increased ROS levels can cause oxidative stress that can damage biomolecules, such as proteins and DNA. A moderate level of oxidative stress can induce the proliferation and metastasis of cancer cells, whereas extremely high oxidative stress is cytotoxic for cancer cells [124,125]. Moreover, a condition known as “reductive stress”, in contrast with “oxidative stress”, characterized by a strong increase in GSH/GSSG, NADPH/NADP^+^ and NADH/NAD^+^ levels or the overexpression of antioxidant enzymes, can deplete ROS and lead to cancer cell survival, resistance to cancer therapy and stemness of cancer cells. This suggests that tumors try to adapt to the high threshold of ROS by shifting their microenvironment to more reductive conditions [126].

The pentose phosphate pathway (PPP) plays a critical role in redox balance since it is the principal source of the reducing agent NADPH, which is crucial for the detoxification of cellular oxidative stress via the conversion of oxidized glutathione into reduced glutathione. 

A deficiency of transaldolase (TAL), a key enzyme in the reversible non-oxidative phase of the PPP, induces hepatocarcinogenesis in mice, as both accumulation of sedoheptulose 7-phosphate and failure to recycle ribose 5-phosphate for the oxidative PPP decrease NADPH and glutathione levels [127]. Interestingly, TAL-deficient mice overexpress NADPH-dependent aldose reductase (AR). Therefore, the large depletion of NADPH in TAL-deficient mice may also be due to the activation of AR, which converts accumulated sugars into polyols consuming NADPH. The resulting NAPDH depletion and oxidative stress predispose the mice to hepatocarcinogenesis.

Recently, Oaks et al. [128] demonstrated that the simultaneous inactivation of TAL and AR in DKO mice increases the NADPH level and blocks mitochondrial oxidative stress and polyols accumulation, thus, preventing the progression from cirrhosis to hepatocellular carcinoma (HCC). The AR inhibitors Zopolrestat and Sorbinil arrest the proliferation of hepatoma and breast cancer cells in vitro. This suggests that AR is a regulatory checkpoint of mitochondrial reactive oxygen intermediate (ROI) production and a critical trigger of hepatocarcinogenesis. Moreover, AR inactivation promotes S7P accumulation and modulates the carbon flux between the PPP and TCA cycle. 

Several studies suggest that elevating ROS production and reducing antioxidant activity may induce cancer cell death [129,130]. Drug-resistant cells often show higher glutathione (GSH) levels compared to non-cancer cells. Therefore, a cancer treatment based on a reduction in GSH content may be appropriate to prevent cancer resistance [131].

The natural compound Phenethylisothiocyanate (PEITC) induces apoptosis in ovarian, osteosarcoma and cholangiocarcinoma cancer cells via GSH depletion [132,133,134]. The inhibitors of GSH synthesis Dimethylaminomicheliolide and Buthionine sulfoximine cause GSH depletion, increased ROS production, mitochondrial dysfunction, and apoptotic cell death in leukemia and glioblastoma cells [135]. Imexon, a chemotherapeutic drug that disrupts the activity of GSH by binding to its thiol group, depletes the GSH pool, inducing a loss of Δψ, an increased ROS level and, finally, apoptotic cell death in human pancreatic cancer cell lines [136].

Various plant dietary compounds, such as polyphenols, are known to act as antioxidants. However, at high concentrations they can function as potential pro-oxidants because, in the presence of copper ions, they increase ROS levels through a Fenton-like reaction [137] and trigger apoptosis in cancer cells.

Extracts from Olea europaea leaves show great anticancer activity since they increase ROS and decrease ROS scavenging enzymes leading to mitochondrial dysfunction and, finally, apoptosis in ovarian cell lines [138].

Sesamol, a phenolic compound present in the seeds of sesame, at high concentration, produce intracellular superoxide in human colon cancer HCT116 cells, leading to mitochondrial dysfunction, DNA fragmentation and apoptosis [139].

The natural flavonoid Baicalein increases intracellular ROS levels in the human breast cancer cell MCF-7, disrupting the mitochondrial membrane and triggering the mitochondrial-dependent apoptotic pathway [140].

In different cancer cells, Curcumin increases ROS production and activates the intrinsic pathway of apoptosis mediated by the induction of mitochondrial dysfunction [141,142].

The use of ROS inducers and antioxidant blockers can provoke side effects, especially in normal cells; therefore, it is crucial to improve the specificity and efficiency of these agents towards tumor cells. Moreover, polyphenols have poor bioavailability and selectivity; therefore, it is necessary to target these compounds to mitochondria by conjugating them with other molecules or via the development of polyphenol-encapsulated micelles [141].

**Figure 3:**
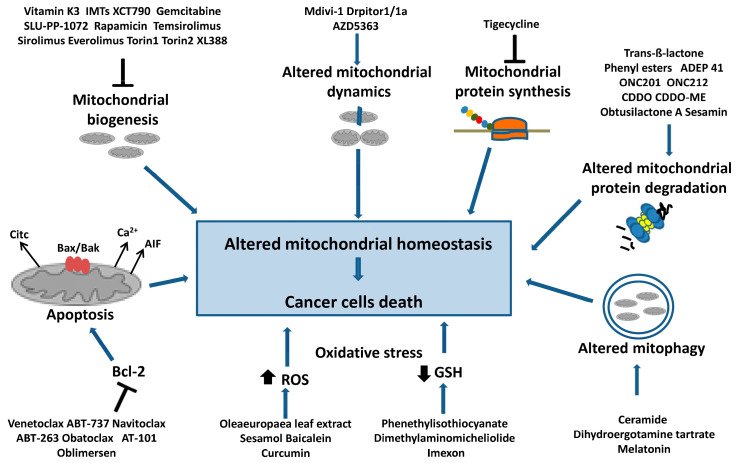
Targeting mitochondrial homeostasis in cancer cells. Are indicated the compounds that cause mitochondrial dysfunction and cancer cell death. In particular, they are: inhibitors of mitochondrial biogenesis that act on mtDNA replication and transcription or on the PGC1-α axis; inhibitors of mitochondrial protein synthesis; molecules inducing the deregulation of mitochondrial protein degradation, dynamics and mitophagy. Additionally, inhibitors of antiapoptotic factor Bcl-2 are shown, as well as drugs inducing intensive oxidative stress through a decrease in the GSH level and an increase in ROS levels, which can cause cancer cell apoptosis.

Figure 3 summarizes the main mechanisms of mitochondrial homeostasis in tumor cells and the best-known molecules that target these pathways and induce cell death.

The drugs targeting mitochondrial homeostasis, discussed in previous paragraphs, are presented in Table 2. The molecular targets and the anti-tumor effect of these molecules are also reported.

## 4. Photodynamic, Photothermal and Chemodynamic Therapies

Photodynamic therapy (PDT) is a locally targeted therapy that uses a photosensitizer (PS), light, and oxygen to selectively kill cancer cells [143]. PS first absorbs photons, then changes from a ground state to a singlet-excited state, and then to a triplet-excited state. Finally, energy is transferred to nearby oxygen molecules in the cells to generate a superoxide anion (type I reaction) or singlet-oxygen (type II reaction). A ROS increase induces mitochondrial damage and the initiation of apoptosis. The most represented PSs are hematoporphyrin derivatives, transition-metal complexes and phthalocyanine. Due to the negative potential of the inner mitochondrial membrane, PSs can be linked to a lipophilic and cationic group, such as an organic phosphine/sulfur salt (TPP) or QA salts (rhodamine and rhodamine derivatives, pyridinium), to specifically target the mitochondria [144]. Many photosensitizers (Photofrin, Foscan, Talaporfin, and Temoporfin) have been used in numerous clinical trials [145,146]. However, this therapy has some limitations, such as low-light-penetration depths, non-targeting photosensitizers, increased GSH levels in tumor cells and tumor hypoxia. Recently, new photosensitizer nanoparticles (TPEPy-BA) were developed, which improve PDT efficacy by increasing mitochondria-targeting and glutathione-depletion capability [147].

Photothermal therapy (PTT) kills cancer cells with thermal damage (conversion of light energy into heat) by utilizing an external light source and a photothermal agent as heat-generating source. The localized heat produces heat damage to the mitochondria and, consequently, to cancer cells [143].

Chemodynamic therapy (CDT) is a new type of therapy. It converts endogenous H_2_O_2_ by the Fenton reaction into a cytotoxic hydroxyl radical that damages important macromolecules and induces apoptosis in tumor tissues [148]. Cancer cells have a much higher level of H_2_O_2_ than normal cells; however, some CDT drugs work by increasing H_2_O_2_ production to enhance oxidative damage [149]. These formulations include the enzyme glucose oxidase (GOD) to produce H_2_O_2_ as well as the iron source for the Fenton reaction. A major limitation of CDT is the increased GSH level and hypoxia of tumor cells, which may reduce therapeutic efficacy. However, strategies aimed at favoring GSH depletion may improve the efficacy of this therapy [150].

## 5. How to Target Mitochondrial Drugs

The permeability of the two mitochondrial membranes is different. The outer mitochondrial membrane can be easily traversed, since it is more permeable, but it is difficult for many therapeutic molecules to cross the inner mitochondrial membrane.

In mitochondria, there is a significant transmembrane potential (negative inside) that may be used to transport positively charged molecules into mitochondria. To deliver drugs into mitochondria, they should be conjugated with positively charged mitochondria-targeting carriers, such as positively charged peptides or lipophilic cations (triphenylphosphonium, rhodamine, pyridinium salts) [151,152]. Triphenylphosphonium salts are the most widely used as they have lipophilic and hydrophilic features, minimal reactivity, in vivo stability, and an absence of light absorption and fluorescence [153].

In recent years, various nanoformulations have been designed to target drugs in mitochondria, such as liposomes, nanomicelles, dendrimers, and nanoparticles [154]. These systems decrease the drug’s size, favoring tumor penetration and lowering the toxicity to normal tissues. These nanoformulations can be administrated in different ways, such as oral, via injectables, and by pulmonary inhalation. These nanoformulations can be conjugated with lipophilic cations, such as rhodamine or triphenylphosphonium, to deliver drugs, preferentially, to the mitochondria of cancer cells. The positively charged nanoformulations can bind the negatively charged phospholipids and then fuse with the cell membrane; thus, forming the endolysosome. When the contents are released into the cytoplasm, the intracellular targeting of the mitochondria takes places.

Liposomes are constituted by bilayer vesicles formed by phospholipids dispersed in water, which are generally biocompatible and nontoxic; moreover, they can carry large drugs [155].

Nanomicelles are amphiphilic molecular systems of spherical shape that contain a hydrophobic core and a hydrophilic environment; therefore, can transport hydrophilic and hydrophobic drugs [156].

Dendrimers are macromolecules consisting of a central core and multiple branches that have various ligands attached to their peripheries [157].

Nanoparticles (NP) can be classified into polymeric and inorganic [158]. Polymeric NPs are sub-micron colloidal systems prepared by binding a copolymer to a polymer matrix. Polymers can be poly (lactic-co-glycolic acid) (PLGA), poly (glycolic acid) (PGA), poly (lactic acid) (PLA), and polycaprolactone (PCL); all biodegradable and biocompatible. They can be classified into nanospheres and nanocapsules: in the former, the drug is dispersed in a matrix; in the second, the drug is encapsulated by a polymeric membrane [159]. Inorganic nanoparticles consist of inorganic components, such as graphene oxide, platinum, gold, silica, iron and carbon; they have good stability and a high drug-loading capacity [158,160]. Inorganic NPs are valuable in that they enhance the treatment efficacy of techniques such as photodynamic and photothermal therapy. However, their clinical use is strictly controlled because of the potential toxicity and low immunogenicity [160].

## 6. Conclusions and Future Prospective

Several studies support the key role of mitochondria in cancer development and progression, although many aspects, as well as their complex metabolic relationships, remain to be understood. Mitochondria play a crucial role in cancer cells not only for energy production but also for many processes that are known to be altered in cancer cells, from metabolism to oxidative stress to apoptosis. Therefore, targeting mitochondrial functionality in cancer cells using specific target inhibitors has emerged as a promising anticancer strategy with high therapeutic opportunities.

Mitochondria-targeted therapy is an alternative strategy for the treatment of numerous cancer types; however, this approach still has some limitations, which are summarized here. First, a better understanding of the differences in mitochondrial function between cancer cells and normal cells is needed. Then, there are several problems related to the efficacy of these mitochondria-targeted drugs, such as their limited capacity to cross the inner mitochondrial membrane and the fact that some of these were developed for a metabolic context in which mitochondrial respiration is the predominant source of energy. A further concern regards the safety and stability of these mitochondria-targeted drugs, in that some of them tend to agglomerate and form precipitates when injected intravenously, with the risk of thrombosis. Finally, racial ethnicity should also be considered as some races may respond better to mitochondrial inhibitors.

Moreover, the crosstalk between the mitochondrion and the nucleus plays a key role in the regulation of several cellular functions, such as the differentiation and adaptation to stress. Mitochondria continuously update the nucleus on their bioenergetic status (retrograde signaling) by producing energy metabolites (mitostress signals). The nucleus responds via the activation of stress response signaling pathways to adjust ATP production to the cell’s energy requirements [161]. The perturbation of nuclear–mitochondria communication could, therefore, represent a new promising target in cancer therapy. Unfortunately, this topic has been little studied. Recently, Zhang et al. [162], analyzing 11 anticancer drugs, reported an anticancer drug mechanism that, by increasing the steady-state levels of ROS, targets a nucleus-to-mitochondria ROS-sensing pathway. Specifically, this pathway involves checkpoint kinase 1 (CHK1) and mitochondrial single stranded DNA-binding protein 1 (SSBP1), and couples the DNA damage response to the control of mitochondrial translation. There are reasons to state that this topic should be better explored.

Many mitochondria-targeted drugs have shown interesting results in preclinical trials, some have already been approved by the FDA as anticancer agents. The integration of drugs acting via mitochondrial destabilization, biomaterials and nanotechnology may provide a potential advantage for precision medicine in cancer; however, several clinical and preclinical studies are still needed. In addition, it can be envisioned that mitochondria-targeted anticancer therapy may also open new avenues in the treatment of mitochondria-related diseases other than cancer.

## Figures and Tables

**Table 1:** Drugs targeting mitochondrial metabolism.

Drugs	Targets	Effects	Ref.
Krebs cycle			
Ivosidenib, Vorasidenib	Mutant IDH	Increase remission and survival in patients with leukemia and glioma	[13,14]
Fatty acid oxidation			
Etomoxir, ST1326, Perhexiline	CPT-1	Prevent the proliferation of glioblastoma, glioma, leukemia and colorectal cancer cells	[19,20,21,22,23]
Glutamine metabolism			
V-9302	Glutamine transporter (ASCT2)	Reduces the proliferation of lung, breast and colorectal cancer cells	[25]
CB-839, BPTES, UPGL00004	GLS	Decrease the proliferation of triple-negative breast and colorectal cancer cells	[26,27]
Propylselen	GDH	Decreases the proliferation of liver and lung cancer cells	[28]
Hydralazine hydrochloride, Aspulvinone O	GOT1	Inhibit the proliferation of colorectal, breast and pancreatic cancer cells	[29,30]
Aminooxyacetate, Oxamate	GPT2	Suppress the proliferation of breast, lung, colon and pancreatic cancer cells	[31,32]
OXPHOS			
Metformin, Phenformin, BAY 87-2243, Fenofibrate, Ivermectin, IACS-010759, Magnolia extracts	Complex I	Antitumor activity in osteosarcoma, neuroblastoma, melanoma, glioblastoma, myeloid leukemia, gastric, breast, ovarian, endometrial, pancreatic, liver, lung, renal, colorectal, brain and oral cancers	[37,38] [44,45,46,47,48,49,50,51,52]
Gracillin, Lonidamine	Complex II	Inhibit the proliferation of melanoma, brain and lung cancer cells	[53,54,55]
Atovaquone	Complex III	Reduces tumor proliferation in pancreatic, breast and lung cancer cells	[56]
Arsenic trioxide	Complex IV	Reduces tumor proliferation in breast and promyelocytic leukemia cancer cells	[57,58]
Bedaquiline, Aurovertin B, Gboxin	Complex V	Induce apoptosis in breast and glioblastoma cancer cells	[59,60,61]
Heme biosynthesis			
Succinylacetone	ALA-D	Reduces growth in leukemic and colon cancer cells	[65,66,67]
Cyclopamine tartrate	ALAS1	Suppresses growth in non-small cell lung cancer (NSCLC)	[68]

**Table 2:** Drugs targeting mitochondrial homeostasis.

	Drugs	Targets	Effects	Ref.
Biogenesis				
	Vitamin K3,	mtDNA polymerase γ	Induces apoptosis in colon, lung, breast, pancreatic, prostate, leukemic cancer cells	[69]
	IMTs	mtRNA polymerase	Induce an antitumor response in xenografts of human cancer cells	[70]
	XCT790, Gemcitabine, SLU-PP-1072	ERRα	Anticancer effects in acute myeloid leukemia, pancreatic and prostate cancer	[77,78,79]
	Rapamycin, Temsirolimus, Sirolimus, Everolimus Torin1, Torin2, XL388	mTOR	Anticancer effects in renal cancer, glioblastoma, glioma, breast and endometrial cancer, lymphomas, multiple myeloma	[81,82,83,84]
Apoptosis				
	Venetoclax, ABT-737, Navitoclax, ABT-263, Obatoclax, AT-101, Oblimersen	Bcl-2	Tumor regression in chronic lymphocytic leukemia, acute myeloid leukemia, breast cancer, gastric cancers, small-cell lung cancer, head and neck squamous cancer	[87,88,89,90]
Dynamics				
	Mdivi-1, Drpitor1/1a	Drp1	Reduce proliferation in lung, breast, pancreatic, thyroid cancer cells	[94,96]
	AZD536	Mfn1	Reduces proliferation in breast cancer cell lines	[97]
Mitophagy				
	Ceramide	BNIP3	Reduces proliferation in glioma cell line	[100]
	Dihydroergotamine tartrate Melatonin	PINK1/Parkin	Induce lung and cervical cancer cell death	[101,102]
Proteostasis				
Protein synthesis	Tigecycline	28S subunit	Slows proliferation in acute myeloid leukemia, gastric, glioma cancer cells	[103]
Protein degradation	Trans-ß-lactone, Phenyl esters	ClpP (inhibition)	Induce apoptosis in AML, osteosarcoma, and liver cell lines	[108,109,110]
	ADEP 41, ONC201, ONC212	ClpP (activation)	Induce apoptosis in kidney and cervical cancers and in osteosarcoma, neuroblastoma, leukemia, and lymphoma cell lines	[108,111,112,113]
	CDDO, CDDO-ME, Obtusilactone A, Sesamin	Lonp1	Induce apoptosis in colon, liver, breast, and lung cancer cells	[117,118]
Redox balance				
	Phenethylisothiocyanate, Dimethylaminomicheliolide, Buthionine sulfoximine, Imexon	GSH	Induce apoptosis in ovarian cancer, osteosarcoma, cholangiocarcinoma, leukemia, glioblastoma, and pancreatic cancer cells	[132,133,134,135,136]
	Olea europaea leaf extract, Sesamol, Baicalein, Curcumin	ROS	Show antiproliferative and pro-apoptotic activity in ovarian, colon, pancreatic, breast, gastric, and glioma cancer cells	[138,139,140,141,142]

## Data Availability

Not applicable.
